# Effect of treatment interruptions and outcomes in cancer patients undergoing radiotherapy during the first wave of COVID-19 pandemic in a tertiary care institute

**DOI:** 10.1186/s43046-022-00129-0

**Published:** 2022-07-04

**Authors:** Sandip Kumar Barik, Arvind Kumar Singh, Minakshi Mishra, Adhar Amritt, Dinesh Prasad Sahu, Saroj Kumar Das Majumdar, Dillip Kumar Parida

**Affiliations:** 1 Department of Radiotherapy, All India Institute of Medical Sciences, Sijua P.O Patrapada, Bhubaneswar, Odisha 751019 India; 2 Department of Community Medicine and Family Medicine, All India Institute of Medical Sciences, Bhubaneswar, Odisha India

**Keywords:** COVID-19, Treatment interruption, Treatment outcomes, Radiotherapy, Cancer

## Abstract

**Introduction:**

COVID-19 patients with cancer had poorer outcomes due to immunosuppression during cancer care, poor general condition, and other comorbidities. The study was conducted to present the real-world analysis of the effect of treatment interruptions on the outcomes of patients treated with radiation therapy during the first wave of the COVID-19 pandemic in a tertiary care institute in India.

**Materials and methods:**

The study is a retrospective observational cohort study on cancer patients undergoing radiation therapy from March 2020 to January 2021. The study’s primary outcome was to analyze the effect of treatment interruptions on the outcomes of patients treated with radiation therapy during the first wave of COVID-19 pandemic.

**Results:**

Between March 2020 to January 2021, 218 eligible patients undergoing radiation therapy were found for the study. Among the 218 patients, 25 patients (11.47%) were found positive for COVID-19, while 193 patients (88.53%) were negative for COVID-19. Among COVID-19-positive patients, ten patients had < 3 weeks of treatment interruption, while 15 patients had > 3 weeks of treatment interruptions. After recovering from COVID-19, treatment was resumed and completed for 15 (60.00%) of the COVID-19-positive patients. In comparison, 13 patients (52.00%) were lost to follow-up. Three of the COVID-19-positive patients died. The disease was clinically controlled in 12 (48.00%) of the COVID-19-positive patients, and the patients reported locoregional disease progression in 10 (40.00%). Among the 193 COVID-19-negative patients, 32 patients (16.58%) had treatment interruption. Twelve patients (37.50%) had treatment interruptions for less than 1 week. There was a significant difference in the delay of radiation treatment delivery by 2 weeks (11 fractions) in COVID-19-positive patients compared to only two fractions delay in COVID-19-negative patients.

**Conclusion:**

COVID-19 impacted the treatment outcomes in both COVID-19-positive and COVID-19-negative cohorts of patients. There was a longer duration of treatment interruptions in the COVID-19-positive patients, leading to fewer patients completing the radiation treatment and thereby increased locoregional disease progression. There was a significant difference in the delay in treatment between the two groups.

## Background

The COVID-19 pandemic poses a significant threat to patients and health care workers. Patients with cancer are at a higher risk of infection with severe acute respiratory syndrome coronavirus-2 (SARS-COV-2) than the general population [1, 2]. Studies have confirmed that COVID-19 patients with cancer had poorer outcomes due to immunosuppression during cancer care, poor general condition, and other comorbidities [3–5]. The radiation oncology departments in a highly populated country like India are taxed by a high caseload on radiotherapy machines, with many patients in the waiting rooms. The waiting areas for cancer patients and treatment areas have to be a highly screened sanctuary to prevent possible transmission and infection of COVID-19 so that both the patients and health caregivers are in a safe and protected environment. The testing of both symptomatic and asymptomatic cancer patients undergoing radiation therapy during the current pandemic is of paramount importance for safely completing treatment. Identifying asymptomatic carriers can help prevent virus transmission to their older, poorer performance status counterparts and other patients with comorbidities. These measures also ensure that cancer patients’ outcomes do not significantly worsen during the pandemic. India reported its first case of COVID-19 on January 30, 2020 [6]. By March 2020, the Government of India laid a series of nationwide lockdowns with severe restrictions applied on interstate and intrastate travel [7]. On 25 August 2021, India have reported 3,25,57,729 confirmed cases of COVID-19,with 436,396 deaths [8]. These directly impacted patients undergoing radiation therapy treatment, which was interrupted as they were infected with COVID-19 or were unable to reach the radiotherapy center for timely completion of treatment. After almost one and half years of the pandemic, the focus is slowly shifting to studying the impact of COVID-19 on cancer patients and their disease outcomes. The study was conducted to present the real-world analysis of the effect of treatment interruptions on the outcomes of patients treated with radiation therapy during the first wave of the COVID-19 pandemic in a tertiary care institute in India.

## Methods

### Study design

The study is a retrospective observational cohort study on cancer patients undergoing radiation therapy at All India Institute of Medical Sciences (AIIMS), Bhubaneswar, a tertiary health care institute in Odisha, India, from March 2020 to January 2021. The study was done with the collaboration of the Department of Radiotherapy and Department of Community Medicine and Family Medicine, AIIMS Bhubaneswar.

### Study outcomes

The primary outcome of the study was conducted to analyze the effect of treatment interruptions on the outcomes of patients treated with radiation therapy during the first wave of the COVID-19 pandemic. The treatment outcomes were interpreted as the duration of treatment interruption, percentage of patients lost to follow-up in patients who did not resume treatment after 2 weeks or more of completion of home or hospital isolation from the day of being diagnosed with COVID-19 infection and were considered lost to follow-up, total percentage of patients completing treatment, an d the clinical status of the disease (locoregional controlled or progression).

### Eligibility criteria

The eligibility criteria included all cancer patients who took Radiation Therapy from March 2020 to January 2021, irrespective of COVID-19 status were included in the study.

### Data collection and study methodology

Data were collected from the patient database of the radiotherapy department and analyzed in August 2021. The information that was collected from the patient’s case records included name, age, sex, diagnosis, COVID-19 status, date of becoming COVID-19 status, date of treatment interruptions, date of treatment resume, number of fractions of radiation planned, number of fractions of radiation delivered, whether treatment completed, current disease status, and number of COVID-19 RTPCR tests done on patients. The datasets included two cohorts of cancer patients who were either positive for COVID-19 or negative for COVID-19. Both the cohorts underwent radiation therapy at AIIMS Bhubaneswar. The cohorts were compared for the effect of treatment interruptions on the outcome of cancer in terms of delay in completion of radiation therapy, duration of treatment interruptions, and current status of the disease. Ethical approval was obtained from the institute ethics committee of All India Institute of Medical Sciences, Bhubaneswar. IEC reference no: T/IM-NF/Radioth/21/24. Individual participant consent was not taken as the data were collected retrospectively from the database.

### Statistical analysis

Data were entered in Microsoft Excel 2015 and analyzed using Statistical Packages of Social Sciences (Version 22.0. Armonk, NY: IBM Corp). The categorical variables were presented as percentages, and continuous variables were presented as mean and standard deviation. Unpaired *t*-test was applied for the test of significance between the COVID-19-positive and COVID-19-negative groups, and a *p*-value of less than 0.05 was considered significant.

## Results

**Fig. 1:**
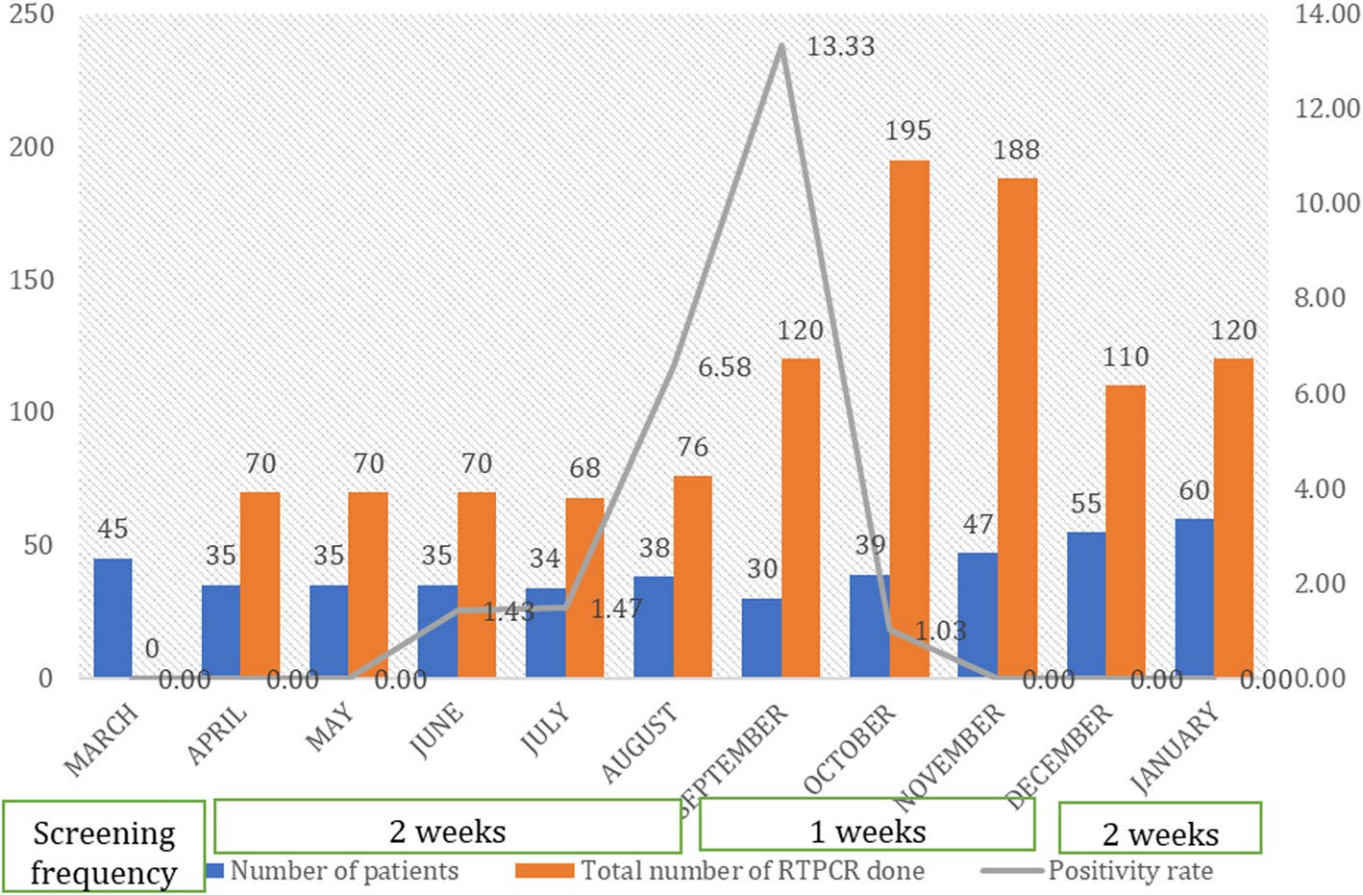
Case positivity rate among the radiotherapy beneficiaries and frequency of screening during March 2020 to January 2021

Between March 2020 to January 2021, 218 eligible patients undergoing radiation therapy were found for the study. The median follow-up time was 8 months. A total of 1087 reverse transcriptase PCR (RTPCR) was done among asymptomatic patients undergoing radiotherapy during this period. The peak positivity rate was a maximum of 13.33 in September 2020. The number of patients screened, the total number of RTPCRs done, and the positivity rate is depicted in Fig. 1.

Among the 218 patients, 25 patients (11.47%) were found positive for COVID-19, while 193 patients (88.53%) were negative for COVID-19. Basic demographic details of the COVID-19-positive and COVID-19-negative patients are mentioned in Table 1. The intent of treatment and the percentage-wise of diagnosis of patients between the COVID-19-positive and COVID-19-negative group has been depicted in Table 2. No significant difference in age and gender was observed between the two groups. Both the groups had radical radiotherapy as the most common intent of treatment. There were no patients in COVID-19-positive patients among the palliative group, while 14 patients (7.25%) in the COVID-19-negative arm. Head and neck cancers, followed by breast cancers, were the most common sites of cancers treated among the two groups.

**Table 1:** Demographic variables of radiotherapy beneficiaries

Variables	COVID-19-positive *N* = 25	COVID-19-negative *N* = 193	*P* value
Total number of patients	25 (11.47%)	193 (88.53%)	
Age (mean ± SD)	45.76 ± 18.87	44.80 ± 16.23	0.786
Gender			
Male	8 (32%)	99 (51.29%)	0.069
Female	17 (68%)	94 (48.70%)	
Treatment interruption	25 (100.00%)	32 (16.58%)

**Table 2:** Intent of treatment and patient diagnosis

**Patients profile**	**COVID-19-positive (*N* = 25)**	**COVID-19-negative (*N* = 193)**
**Types of radiotherapy received**		
Radical radiotherapy	14 (56%)	93 (48.18%)
Adjuvant radiotherapy	11 (44%)	86 (44.55%)
Palliative radiotherapy	0	14 (7.25%)
**Cancer diagnosis of patients**		
Carcinoma head and neck	06 (24%)	59 (30.56%)
Carcinoma breast	09 (36%)	52 (26.94%)
Carcinoma rectum and anal canal	1 (4%)	11 (5.69%)
Carcinoma lung	1 (4%)	2 (1.03%)
Carcinoma esophagus	1 (4%)	5 (2.59%)
Carcinoma cervix	02 (8%)	08 (4.14%)
CNS tumors	03 (12%)	15 (7.77%)
Pediatric tumors	02 (8%)	12 (6.21%)
Others	0	29 (15.02%)

All the patients had treatment Interruptions following the diagnosis of COVID-19 as per guidelines of the Ministry of Health and Family Welfare, India, on mandatory quarantine and home isolation for COVID-19-positive patients [9]. Among COVID-19-positive patients, only ten patients (40%) had < 3 weeks of treatment interruption, while 15 patients (60%) had > 3 weeks of treatment interruptions. Three patients had multiple treatment interruptions as they were reinfected with COVID-19. After recovering from COVID-19, treatment was resumed and completed for 15 (60.00%) of the COVID-19-positive patients. In the COVID-19-positive arm, ten patients (40%), while in the COVID-19-negative arm, six patients (18.75%) were lost to follow-up and did not complete their assigned fractions of radiation therapy. Three of the COVID-19-positive patients died, one due to COVID-19 infection and two due to their disease progression. The disease was clinically controlled in 12 (48.00%) of the COVID-19-positive patients, and the patients reported locoregional disease progression in 10 (40.00%). Two patients developed the metastatic disease, while the status remained unknown for one patient (Table 3).

**Table 3:** Treatment interruption and treatment outcome in COVID-19-positive and COVID-19-negative patients

Variables	COVID-19-positive patients (25)	COVID-19-negative patients (32)	*P* value
Duration of treatment interruption in COVID-19-positive patients			
< 3 weeks	10 (40.00)	12 (37.50)	0.847
> 3 weeks	15 (60.00)	20 (63.50)	
Treatment completion in COVID-19-positive patients			
Total treatment completed	15 (60.00)	26 (81.25)	0.076
Loss to follow-up	10 (40.00)	6 (18.75)	
Treatment outcome in COVID-19-positive patients			
Disease clinically controlled	12 (48.00)	21 (65.62)	
Loco-regional progression of disease	10 (40.00)	3 (9.37)	
Developed metastatic disease^a^	2 (8.00)	1 (3.13)	
Status unknown	1 (4.00)	6 (18.75)	
Death due to other cause^b^	0 (0.00)	1 (3.13)	

^a^Death happened in the COVID-19-negative group

^b^The patient died of road traffic accident

Among the 193 COVID-19-negative patients, 32 patients (16.58%) had treatment interruption. Twelve patients (37.50%) had treatment interruptions for less than 3 weeks (six patients for radiation-induced grade 3 toxicity and six for personal reasons), and 20 patients (63.50%) had treatment interruptions > 3 weeks. Two patients died during treatment, one due to disease progression and one road traffic accident. Three patients (9.37%) had clinical disease progression, and six patients left treatment midway and did not complete the treatment (Table 3).

The COVID-19-positive patients received an average of 16.72 fractions, whereas the negative patients received 21.71 fractions of radiotherapy. Averagely, 10.80 fractions and 2.44 fractions of radiotherapy could not be delivered to the COVID-19-positive and COVID-19-negative patients, respectively, which correlates with a delay of radiation treatment delivery by almost 2 weeks (11 fractions) in COVID-19-positive patients as compared to only two fractions delay in COVID-19-negative patients. The difference was also found to be statistically significant (Table 4).

**Table 4:** Radiotherapy fractions planned and delivered among the radiotherapy recipients (*N* = 218)

Variables	COVID-19-positive patients (mean ± SD) *N* = 25	COVID-19-negative patients (mean ± SD) *N* = 193	*P* value
Radio therapy fractions planned (*N* = 25)	27.52 ± 3.98	28.32 ± 3.68	0.026
Radio therapy fractions delivered (*N* = 25)	16.72 ± 9.14	21.71 ± 10.00	0.019
Radio therapy fractions not delivered (*N* = 25)	10.80 ± 9.57	2.44 ± 7.14	< *0.001*

## Discussion

A departmental screening protocol was developed because of the COVID-19 pandemic, which included screening patients and visitors to the department in consultation with the hospital Infection control committee. All persons were screened for signs, symptoms, travel history, and contact history at the entry point. Detection of SARS-CoV-2 viral RNA by RT-PCR remains the gold standard for diagnosis, preferably with a nasopharyngeal swab [10]. The guidelines formulated for screening patients in an oncology department were strictly followed. Considering the current situation, the policies were changed from time to time, considering the current position of the COVID-19 pandemic.

We started with screening all patients who were to start radiotherapy by RT-PCR testing for COVID-19 virus. During radiotherapy, all patients were followed up weekly, and symptomatic patients were immediately isolated and tested by RT-PCR, while all asymptomatic patients on radiotherapy were tested every month from April 1, 2020, to June 30, 2020. As the COVID-19-positive cases increased from July 1, 2020, to August 31, 2020, all asymptomatic patients were tested every fortnightly, and after that, all patients were tested weekly. According to the Indian Council of Medical Research (ICMR) advisory recommendation on COVID-19 testing in India, in-hospital setting RT-PCR or rapid antigen testing to be done on all symptomatic and severe acute respiratory infection patients. RT-PCR for COVID-19 is recommended In asymptomatic high-risk patients hospitalized or seeking immediate hospitalization, such as immunocompromised individuals, patients diagnosed with malignant disease, transplant patients, elderly > 65 years, and comorbidity undergoing surgical or non-surgical invasive procedures [10, 11].

This approach may be suboptimal for cancer patients undergoing radiation therapy. Most of the patients will be asymptomatic for COVID-19, and the virus’s overall transmission is mainly contributed during the early phase of illness [12]. Hence, screening asymptomatic carrier patients taking radiation therapy becomes more important to protect other vulnerable patients and healthcare workers.

Manchester et al. screened all asymptomatic patients twice a week with RT PCR from April 17, 2020, to May 8, 2020; 139 patients also showed a positivity rate of 0.7% [13]. Another multicenter survey by Vasiliadou et al. on head and neck cancer patients during COVID-19 pandemic revealed that around 52.2% of centers performed pre-treatment swab tests, and about four centers were doing it weekly [14].

Our study found that 25 (11.47%) patients out of 219 patients were positive for COVID-19. This may be due to the longer follow-up period, about 10 months, and the high caseload of COVID-19-positive patients in India.

Patients who become positive during treatment require a minimum of 14 days of quarantine, according to ICMR recommendations [9], resulting in a delay in treatment. Thus, treatment interruptions are of concern in tumors where delay in overall treatment time leads to a poor outcome, such as cancer of the head, neck, and cervix [15]. William et al., in a study on the impact of COVID-19 on treatment, have expressed that a delay in treatment until a negative COVID-19 test may be indicated to protect the patient and the treatment team and to maintain access to care for all the patients treated at that facility [16].

This statement holds good for a cancer center like us with limited recourses and an enormous caseload. In the current study, among the COVID-19-positive patients, 40% had less than 3 weeks while 60% patients had more than 3 weeks of treatment interruptions. Disease control was seen in 48% of COVID-19-positive patients, and 40% had locoregional disease progression, while in the COVID-negative patients’ cohort, 37.50% had treatment interruptions of less than 3 weeks, and 63.50% had treatment interruptions of more than 3 weeks. The main reasons for treatment interruptions in COVID-negative patients beyond 3 weeks were travel restrictions, fear of getting infected with COVID on moving out of home, and any other factors like family issues, job loss, and other social stigmas. The disease was controlled in 65.62% of COVID-negative patients, and 9.37% had disease progression.

The radiation oncology department from Fudan University, Shanghai, China, in their paper on nasopharyngeal carcinoma patients, has compared the median waiting days between pre-COVID onset to that after the onset of the COVID-19 pandemic and has reported a significant difference in results for radiotherapy procedures like radiotherapy immobilization and simulation (3.5 vs. 16.5, *p* < 0.001), radiotherapy planning (20 vs. 61, *p* < 0.001), and initiation of radiation (28 vs. 36, *p* = 0.005) [17]. Riera et al. reported a delay in radiotherapy by 1.4 to 90.9% of centers with an 8 to 45 days median increase compared to pre-COVID times. Treatment interruptions were reported by 77.5% of patients, although they have not specified what type of treatment interruptions the patients have to face [18]. A cohort study from India also mentions the considerable impact of the COVID-19 pandemic on the delivery of oncology services. The percentage reduction in patients accessing oncology services was higher in tier 1 cities than in tier 3 cities, with about 50–70% reduction observed in almost all cancer care services [19]. According to Conghua et al., in a study on analysis of treatment outcomes of 209 patients undergoing radiotherapy at the Wuhan University, 112 patients (56%) defaulted to radiotherapy due to lockdown [20]. In an observational cohort study by the Dutch Oncology COVID-19 Consortium on the outcome of COVID-19 patients with cancer, out of 442 patients, only 30.8% of patients completed treatment. By multivariate analysis, age > 65 years, male gender, prior history of malignancy, and hematological or lung malignancy remained independent risk factors for fatal outcome of COVID-19 [21].

This study is among few kinds of literature available on the impact of COVID-19 on patients undergoing radiation therapy with real-world data analysis of the treatment interruptions and disease progression. However, the study presents data from a single institute, and only a univariate analysis could be done with a small sample size of COVID-19-positive patients. Also, the study did not investigate multiple confounders, including the reason for treatment delay in both COVID-19-positive and COVID-19-negative cohorts.

## Conclusions

Due to COVID-19, treatment interruptions were found in both COVID-19-positive and COVID-19-negative patients. Various policy restrictions were the primary reasons for the treatment interruptions in COVID-19-negative patients. There was a longer duration of treatment interruptions in the COVID-19-positive patients, leading to fewer patients completing the radiation treatment and thereby increased locoregional disease progression. There was a significant difference in the delay of radiation treatment delivery in COVID-19-positive patients compared to COVID-19-negative patients.

## Data Availability

The dataset for the study is available in the following link: Barik, Sandip Kumar (2022): RT DATA COVID 19 Treatment Interruptions.xlsx. figshare. Dataset. https://doi.org/10.6084/m9.figshare.19298624.v1.

## Abbreviations

SARS-COV-2: Severe acute respiratory syndrome coronavirus-2
RTPCR: Reverse transcriptase PCR
AIIMS: All India Institute of Medical Sciences
